# The effectiveness of video-based multiple training modalities in community service centers for dementia: a cluster randomized controlled study

**DOI:** 10.3389/fmed.2025.1540608

**Published:** 2025-06-11

**Authors:** Sun-Gen Yang, Wen-Fu Wang, Yu-Chun Tung, Shang-Chien Huang, Ming-Che Chang, Ling-Chun Huang, Yuan-Han Yang, Kai-Ming Jhang

**Affiliations:** ^1^Department of Medical Education, Changhua Christian Hospital, Changhua, Taiwan; ^2^Department of Neurology, Changhua Christian Hospital, Changhua, Taiwan; ^3^Department of Pharmacy, Taichung Veterans General Hospital, Taichung, Taiwan; ^4^Department of Psychiatry, Tungs’ Taichung MetroHarbor Hospital, Taichung, Taiwan; ^5^Department of Nuclear Medicine, Changhua Christian Hospital, Changhua, Taiwan; ^6^Department of Neurology, Kaohsiung Medical University Hospital, Kaohsiung, Taiwan; ^7^Neuroscience Research Center, Kaohsiung Medical University, Kaohsiung, Taiwan; ^8^Department of Neurology, Kaohsiung Medical University Gangshan Hospital, Kaohsiung Medical University, Kaohsiung, Taiwan; ^9^School of Post-Baccalaureate Medicine, College of Medicine, Kaohsiung Medical University, Kaohsiung, Taiwan

**Keywords:** dementia, cognitive training, physical exercise, caregiver burden, BPSD (behavioral and psychological symptoms in dementia)

## Abstract

**Objectives:**

This study aimed to evaluate the effectiveness of a 16-week video-based multiple training modality (MTM) program for dementia patients in Community Service Centers for Dementia (CSCDs) in Taiwan.

**Design:**

Cluster Randomized controlled trial.

**Setting:**

A total of 16 CSCDs across four counties in Taiwan.

**Participants:**

A total of 207 dementia patients and their caregivers, randomly clustered and assigned to an intervention group or a control group.

**Intervention:**

The intervention group received a 16-week video-based MTM program including dynamic physical exercises and creative activities. The control group continued with regular activities at the CSCDs.

**Measurements:**

Outcomes measured were behavioral and psychological symptoms of dementia (BPSD) using the Neuropsychiatric Inventory Questionnaire (NPI-Q), caregiver burden using the Zarit’s Burden Interview (ZBI), and caregiver depression using the Center for Epidemiologic Studies Depression Scale (CES-D). These were assessed at baseline and post-intervention.

**Results:**

The intervention group showed a significant reduction in appetite/eating distress compared to the control group (change in NPI scores: −0.2 vs. 0, *p* = 0.026). While there was a trend toward reduced caregiver burden, the results were not statistically significant (change in ZBI scores: −3.1 vs. –1.2, *p* = 0.306). No significant changes were observed in overall BPSD severity or caregiver depression.

**Conclusion:**

The video-based MTM program showed potential in improving specific neuropsychological symptoms, especially appetite/eating distress, in dementia patients. The program’s ease of implementation suggests it could be expanded across CSCDs in Taiwan, with a recommendation for refinement to better support caregivers.

## Introduction

Dementia is a degenerative disease that affects memory, judgment, language, and behavior. Its prevalence increases rapidly with age, making it a significant medical issue in developed countries with a high proportion of an elderly population (1). Dementia not only affects memory but also leads to delusions, hallucinations, and disruptive behavior, creating a substantial burden for family caregivers (2–4).

Elderly individuals with dementia, apart from cognitive decline, often lose interest in outdoor or physical activities due to apathy or depression. This disinterest can lead to a faster decline in their physical and global functions (5, 6), leading to conditions such as frailty, sarcopenia, reduced muscle strength, and decreased fitness. Frailty and sarcopenia further affect the quality of life, leading to daily functional disabilities, falls, depression, increased chances of hospitalization, and, in severe cases, even death (7–9). Many scientific studies have confirmed that cognitive and exercise interventions not only help delay cognitive decline in middle-aged and older adults but also improve their physical fitness, increase muscle strength, and reduce bone loss (10–12).

Previous research indicates that multidomain interventions can enhance physical fitness and body function in individuals with dementia (13). Systematic analysis shows that cognitive training can slightly improve cognitive functions in dementia cases (14). We have developed multiple training modalities, combined with exercise and static programs (15, 16), which contain a 16-week dynamic exercise program based on existing literature, focusing primarily on strength training exercises, supplemented with cognitive activities and games that combine cognitive and physical elements. This approach not only offers more mental stimulation but also, through physical fitness training, allows seniors to engage in dynamic games, therefore enhancing their social interactions. The static program includes calligraphy and painting courses. These multiple training modalities have been found to improve physical fitness and the severity of depression in the elderly with subjective memory complaints (15). A small study (24 cases of Alzheimer’s disease) showed improvements in cognitive function, psychiatric symptoms, and reduced caregiver distress in dementia patients (16).

Since the launch of the Taiwan Long-Term Care 2.0 initiative in 2017, which includes dementia patients over the age of 50, innovative service items such as “Dementia Integrated Care Centers” and “Community Service Center for Dementia (CSCD)” have been established (17, 18). “Dementia Integrated Care Centers,” often run by medical institutions in various countries and cities, focus on case management and a co-care platform (19, 20). CSCDs provide cognitive stimulation group courses and respite services for people living with dementia (PLWD). According to the dementia care resources compiled by the Ministry of Health and Welfare, until December 2023, there were 533 CSCDs across Taiwan, serving 17,102 people in 2023 (21). Most cognitive stimulation courses offered at these service centers have not been empirically validated, and care staff lack standardized training about cognitive training.

In order to promote evident-based training programs, we recorded videos of previously validated multiple training modalities. Care staff at CSCD use these videos to conduct training courses. The aim of the study is to elucidate the effectiveness of the video-based multiple training modalities for people living with dementia who were cared for in CSCDs.

## Materials and methods

### Design and participants

This study is a cluster-randomized controlled trial. Participants were people living with dementia cared for in CSCDs. Sixteen CSCDs from four counties (Changhua, Taichung, Hsinchu, and Chiayi), covering north to south Taiwan were recruited. Sixteen CSCDs consisted of eight pairs of experimental and control groups matched based on similar characteristics, including the number of cases joining group training and the severity of dementia. CSCDs with similar characteristics are defined as differences less than 20% in both of the following conditions: (1) the number of cases cared for and (2) the proportion of patients with the global Clinical Dementia Rating Scale equal to 0.5.

Eight pairs of CSCDs are then randomly assigned to experimental and control groups after obtaining informed consent. PLWDs in the CSCDs were excluded if without caregivers, caregivers were unable to complete assessment scales, or they required assistive devices to stand or walk, and those whose participation in CSCD or intervention activities fell below 70% of the time were also excluded. This study was approved by the Institutional Review Board of Changhua Christian Hospital (CCH IRB 230315). All participants signed an informed consent to participate in the study. We confirmed that all methods were performed in accordance with the relevant guidelines.

### Video-based multiple training modalities

During the program, the experimental group will receive a video-based multi-domain exercise cognitive intervention course in addition to their regular activities at the CSCDs. This program lasts for 16 weeks, with two mornings per week, led by care staff in the CSCD. On one morning, a 2-h dynamic session is conducted (a multi-component physical fitness program), and on the other morning, a static session (1 h of calligraphy and 1 h of painting).

The dynamic session includes aerobic activities to warm up the muscles, followed by the main strength-enhancing exercises, and then stretching exercises as a cool-down method. Strength training covers the upper limbs, trunk, and lower limbs, especially using the elderly’s body weight to adjust the intensity for lower limb strength. Each session ends with balance training, which involves shifting the center of gravity while standing on both or one leg and performing dual or multiple tasks (additional movement or cognitive tasks) in dynamic gait scenarios. The focus of balance training is not on enhancing muscle strength but on helping the elderly learn appropriate postural responses in various sensory feedback situations.

The static activity sessions include calligraphy and painting. Sessions start with simple guides from a basic introduction and progress through the course, using mainly ink and paint, starting from template copying to theme-based creation. The focus of training is to help stimulate cognitive and fine motor skills.

### Regular activities in CSCD

CSCDs were supported by the Long-term Care Act 2.0 in Taiwan. CSCDs provide cognitive training or stimulation lessons as well as caregiver support and training lessons specifically for dementia patients and their care partners. Most community dementia care centers arrange daytime small group (usually 6–20 individuals) cognitive training or stimulation programs 3–5 days per week. The cognitive program was designed by multidisciplinary professionals, such as occupational therapists, and operated by care staff in the CSCDs trained in dementia care. The program has five goals to achieve with different classes to delay dementia disability and prevention, including cognitive enhancement, social interaction, muscle strengthening, balance training, and fall risk prevention. Finally, daily living skills maintenance and training. Classes include art, crafts and handcrafts, music class, dance class, board games, simple stretching, and exercises.

### Measurements

The study aimed to evaluate the impact of the video-based multiple training modalities intervention on behavioral and psychological symptoms of dementia (BPSD), caregiver burden, and caregiver depression status. The severity of BPSD was measured using the Neuropsychiatric Inventory Questionnaire (NPI-Q), which was scored by family caregivers and care staff in the CSCDs, respectively. Changes in caregiver burden were assessed using the Zarit’s Burden Interview (ZBI) (22), a widely recognized tool for understanding the stress and strain experienced by caregivers. Additionally, the Center for Epidemiologic Studies Depression Scale (CES-D) (23) was used to evaluate the depression status of caregivers, offering a quantitative measure of their mental health. Furthermore, a satisfaction survey was conducted among site staff to gauge their feedback on the video version of the multifaceted exercise cognitive intervention program. This comprehensive approach ensured a thorough assessment of both participant and caregiver outcomes, as well as the program’s reception by the implementing staff.

### Statistical analysis

All data were analyzed using R software (R Foundation for Statistical Computing). Pearson’s chi-squared test or Fisher’s exact test was used to test for differences in categorical data. Numerical data were tested using the Kruskal–Wallis rank sum test. A linear regression model was performed to adjust baseline scores if a significant group difference was detected. Differences were considered statistically significant when the *p*-value was less than 0.05.

### Data availability

The datasets used and analyzed during the current study available from the corresponding author on reasonable request.

## Results

### Demographic characteristics

The study included 207 participants, with 105 in the control group and 102 in the intervention group. Four participants in the intervention group were excluded because to participated less than 70% of the time. Table 1 shows the baseline demographic characteristics of the participants. There were no significant differences between the groups in terms of sex, age, and baseline NPI-Q scores. The control group had a higher severity of dementia at baseline compared to the intervention group (CDR = 0.5 72% vs. 55.2%, *p* = 0.013). The intervention group’s staff has a lower caregiving burden at baseline compared to the control group (ZBI pretest 6.3 vs. 10.4, *p* = 0.008).

**Table 1-1:** Demographic characteristics between control and intervention groups.

	Control group (*N* = 105)		Intervention group (*N* = 98)		*p*
Male, *n* (%)	22 (21%)		21 (21%)		>0.9
Age, mean (*SD*)	79.5 (7.2)		78.7 (8.0)		0.4
Global CDR, *n* (%) 0.5 1 2 3	58 (55.2%) 38 (36.2%) 7 (6.7%) 2 (1.9%)		71 (72%) 17 (17%) 9 (9%) 1 (1%)		0.013
CDR-SB, mean (SD)	5.2 (4.4), *n* = 69		5.0 (3.9), *n* = 56		0.9
Table 1-2 Baseline BPSD, caregiver burden and depression between groups					
	Control group (*N* = 105)		Intervention group (*N* = 98)		
NPI-Q (pre-test), mean (*SD*)	Family caregiver*^p1^*	Staff in care site^p2^	Family caregiver*^p1^*	Staff in care site*^p2^*	*p1*, *p2*
NPI-Q total scores	4.5 (5.9)	1.8 (2.9)	4.3 (5.4)	2.0 (2.8)	0.800, 0.580
Delusion severity	0.4 (0.7)	0.1 (0.4)	0.3 (0.7)	0.2 (0.5)	0.762, 0.436
Delusion distress	0.5 (1.1)	0.1 (0.5)	0.5 (1.1)	0.2 (0.7)	0.599, 0.364
Hallucination severity	0.2 (0.6)	0.1 (0.3)	0.2 (0.6)	0.1 (0.4)	0.843, 0.347
Hallucination distress	0.3 (1.0)	0.0 (0.2)	0.3 (0.9)	0.1 (0.5)	0.759, 0.106
Agitation severity	0.3 (0.7)	0.1 (0.4)	0.3 (0.6)	0.1 (0.5)	0.671, 0.717
Agitation distress	0.5 (1.1)	0.1 (0.6)	0.4 (1.0)	0.1 (0.5)	0.703, 0.838
Depression severity	0.7 (0.9)	0.4 (0.8)	0.6 (0.8)	0.4 (0.6)	0.488, 0.434
Depression distress	0.7 (1.2)	0.4 (0.9)	0.6 (0.8)	0.2 (0.5)	0.254, 0.077
Anxiety severity	0.6 (0.9)	0.3 (0.7)	0.5 (0.8)	0.3 (0.6)	0.598, 0.525
Anxiety distress	0.7 (1.3)	0.2 (0.7)	0.5 (0.9)	0.3 (0.7)	0.257, 0.631
Euphoria severity	0.2 (0.5)	0.0 (0.1)	0.2 (0.5)	0.1 (0.3)	0.937, 0.192
Euphoria distress	0.2 (0.6)	0.0 (0.1)	0.1 (0.5)	0.0 (0.1)	0.657, 0.984
Apathy severity	0.5 (0.9)	0.3 (0.7)	0.4 (0.7)	0.1 (0.4)	0.210, 0.071
Apathy distress	0.6 (1.1)	0.3 (0.7)	0.4 (1.0)	0.1 (0.3)	0.310, 0.2
Disinhibition severity	0.3 (0.7)	0.1 (0.4)	0.3 (0.7)	0.2 (0.4)	0.995, 0.393
Disinhibition distress	0.4 (0.9)	0.1 (0.5)	0.4 (1.1)	0.2 (0.5)	0.936, 0.545
Irritability severity	0.4 (0.8)	0.2 (0.5)	0.5 (0.7)	0.3 (0.6)	0.445, 0.232
Irritability distress	0.5 (1.1)	0.2 (0.6)	0.6 (1.1)	0.3 (0.7)	0.537, 0.269
Aberrant motor severity	0.3 (0.7)	0.1 (0.3)	0.4 (0.7)	0.2 (0.5)	0.550, 0.063
Aberrant motor distress	0.4 (0.9)	0.1 (0.4)	0.5 (1.1)	0.1 (0.5)	0.566, 0.178
Nighttime behavior severity	0.4 (0.8)	0.0 (0.3)	0.3 (0.7)	0.0 (0.1)	0.399, 0.160
Nighttime behavior distress	0.5 (1.1)	0.0 (0.2)	0.4 (1.1)	0.0 (0.0)	0.530, 0.181
Appetite/eating severity	0.3 (0.6)	0.1 (0.3)	0.4 (0.7)	0.1 (0.3)	0.329, 0.790
Appetite/eating distress	0.3 (0.7)	0.0 (0.2)	0.4 (0.9)	0.1 (0.3)	0.342, 0.266
ZBI, pre-test	21.8 (17.6)	10.4 (12.1)	21.6 (17.0)	6.3 (8.5)	>0.9, 0.008
CESD, pretest	12.7 (10.0)		12.2 (9.1)		0.8

No significant differences between groups about sex, age, and baseline NPI-Q scores. Control group had severer dementia stage and higher baseline care burden scored by staff in care sties.

### Behavioral and psychological symptoms of dementia (BPSD)

Table 2 shows the changes in various neuropsychological symptoms after 16 weeks of intervention. Reduction in appetite/eating distress compared to the control group was reported by family caregivers in the intervention group (−0.2 vs. 0, *p* = 0.026) after video-based MTM use. There were no significant differences between the control and intervention groups in terms of other symptom severity and distress.

**Table 2:** Changes in neuropsychiatric inventory (NPI) scores between groups.

	Control (*N* = 105)		Intervention (*N* = 98)		*p^a^*
	Family caregiver*^p1^*	Staff in care site*^p2^*	Family caregiver*^p1^*	Staff in care site*^p2^*	*p1, p2*
NPI-Q total scores	Mean (SD)				
Pre-test	4.5 (5.9)	1.8 (2.9)	4.3 (5.5)	0.7, 0.4	0.7, 0.4
Post-test	3.6 (5.6)	1.4 (2.0)	3.9 (5.2)	0.6, 0.5	0.6, 0.5
Difference	−0.9 (5.9)	−0.3 (1.9)	−0.4 (4.2)	0.5, 0.5	0.5, 0.5
NPI-Q subscore differences					
Delusion severity	−0.0 (0.7)	−0.0 (0.4)	0.1 (0.7)	−0.1 (0.4)	0.525, 0.685
Delusion distress	−0.0 (1.0)	−0.1 (0.5)	0.0 (0.9)	−0.1 (0.6)	0.564, 0.521
Hallucination severity	0.0 (0.7)	0.0 (0.4)	0.1 (0.6)	0.0 (0.4)	0.475, 0.474
Hallucination distress	−0.0 (1.0)	0.0 (0.3)	0.1 (0.8)	−0.1 (0.6)	0.301, 0.280
Agitation severity	−0.1 (0.7)	−0.0 (0.3)	−0.0 (0.6)	−0.0 (0.5)	0.281, 0.700
Agitation distress	−0.2 (1.1)	−0.1 (0.4)	−0.0 (1.1)	−0.0 (0.5)	0.436, 0.668
Depression severity	−0.1 (0.8)	−0.1 (0.6)	−0.0 (0.7)	−0.2 (0.7)	0.494, 0.441
Depression distress	−0.1 (1.2)	−0.1 (0.5)	−0.0 (0.9)	−0.1 (0.6)	0.526, 0.669
Anxiety severity	−0.2 (0.7)	0.0 (0.6)	−0.2 (0.6)	0.0 (0.6)	0.983, 0.913
Anxiety distress	−0.2 (1.1)	−0.0 (0.5)	−0.2 (0.8)	−0.0 (0.8)	0.511, 0.767
Euphoria severity	−0.1 (0.5)	0.0 (0.2)	0.0 (0.6)	−0.0 (0.3)	0.167, 0,479
Euphoria distress	−0.1 (0.5)	0.0 (0.1)	0.0 (0.8)	0.0 (0.3)	0.174, 0.562
Apathy severity	−0.1 (0.8)	−0.1 (0.6)	−0.1 (0.8)	−0.0 (0.4)	0.638, 0.170
Apathy distress	−0.1 (1.0)	−0.1 (0.6)	−0.1 (1.0)	0.0 (0.3)	0.969, 0.02
Disinhibition severity	−0.0 (0.7)	0.0 (0.3)	0.0 (0.7)	0.0 (0.6)	0.712, 0.875
Disinhibition distress	−0.0 (1.0)	0.0 (0.3)	−0.0 (1.1)	−0.0 (0.7)	0.801, 0.684
Irritability severity	−0.0 (0.8)	−0.1 (0.5)	−0.1 (0.6)	−0.1 (0.7)	0.601, 0.613
Irritability distress	−0.0 (1.2)	−0.1 (0.6)	−0.2 (0.9)	−0.1 (0.8)	0.396, 0.941
Aberrant motor severity	−0.1 (0.6)	−0.0 (0.2)	0.0 (0.7)	−0.1 (0.3)	0.344, 0.317
Aberrant motor distress	−0.1 (0.9)	−0.0 (0.2)	−0.0 (0.9)	−0.0 (0.5)	0.601, 0.973
Nighttime behavior severity	−0.1 (0.8)	0.0 (0.4)	−0.0 (0.6)	0.0 (0.4)	0.246, 0.557
Nighttime behavior distress	−0.2 (1.2)	0.0 (0.4)	−0.1 (1.0)	0.1 (0.4)	0.514, 0.697
Appetite/eating severity	−0.0 (0.6)	−0.0 (0.3)	−0.2 (0.6)	−0.0 (0.3)	0.054, 0.622
Appetite/eating distress	0.0 (0.8)	0.0 (0.4)	−0.2 (0.8)	−0.0 (0.4)	0.026, 0,323

^a^Kruskal–Wallis rank sum test. A significant reduction in appetite/eating distress after 16 weeks of video-based multiple training modalities compared to the control group.

### Caregiver burden and depression

Table 3 shows the changes in ZBI score from the initial assessment and after intervention. No significant difference was found in ZBI score (−3.1 vs. –1.2, *p* = 0.306 in family caregivers; 3.4 vs. 3.0, *p* = 0.188 in staff at the care site). Although there is no significant difference in ZBI score, a lowering of ZBI score in family caregivers in the intervention group is noticed. No significant difference was found in CES-D scores between the intervention and control group (−0.1 vs. –1.2, *p* = 0.7).

**Table 3:** Changes in care burden and caregiver depression between groups.

	Control (*N* = 105)		Intervention (*N* = 98)		*p^a^*
	Family caregiver*^p1^*	Staff in care site*^p2^*	Family caregiver*^p1^*	Staff in care site*^p2^*	*p1, p2*
ZBI, pre-test	21.8 (17.6)	10.4 (12.1)	21.6 (17.0)	6.3 (8.5)	>0.9, 0.008
ZBI, post-test	20.6 (18.2)	13.5 (9.5)	18.5 (17.1)	9.8 (14.6)	*0.4*, <0.001
ZBI, difference	−1.2 (14.2)	3.0 (7.8)	−3.1 (13.0)	3.4 (17.0)	0.306, 0.188^b^
CESD, pre-test	12.7 (10.0)	–	12.2 (9.1)	–	0.8
CESD, post-test	11.5 (9.1)	–	12.1 (8.4)	–	0.5
CESD, difference	−1.2 (8.2)	–	−0.1 (7.5)	–	0.7

^a^Kruskal–Wallis rank sum test. ^b^Linear regression model to adjust the pre-test ZBI was performed. No significant differences in caregiver burden and depression between groups post-intervention, though it noted a trend toward reduced burden among family caregivers in the intervention group.

## Discussion

The 16-week intervention program demonstrated several notable benefits for both dementia patients and their caregivers. Participants in the intervention group exhibited a significant reduction in specific neuropsychological symptoms, particularly in appetite/eating distress as reported by family caregivers. Additionally, there was a noticeable trend toward a reduction in caregiver burden among family caregivers, although this difference did not reach statistical significance.

The impact of the intervention on Behavioral and Psychological Symptoms of Dementia (BPSD) was evident in the reduction of appetite/eating distress. The structured and engaging nature of the program, which included dynamic exercises, calligraphy, and painting, likely contributed to this improvement. Physical exercise can positively influence appetite in dementia patients through several mechanisms. Blundell et al. (24) explored the interaction between physical activity and appetite control, suggesting that physical activity can stimulate appetite by enhancing metabolic processes and improving overall physiological function. Their findings indicate that engaging in regular physical exercise may increase hunger and food intake by modulating appetite-related hormones and increasing energy expenditure (24). Additionally, Blundell et al. (2015) reviewed the impact of exercise on appetite control and energy balance, highlighting that exercise promotes a positive energy balance by stimulating appetite and enhancing energy intake, which can be particularly beneficial for elderly individuals experiencing reduced appetite (25). Buchman et al. (2012) further supported these findings by demonstrating that higher levels of daily physical activity are associated with reduced risks of cognitive decline and Alzheimer’s disease in older adults (26). This suggests that regular physical activity not only supports cognitive health but also contributes to maintaining overall physiological wellbeing, which can indirectly support appetite regulation in dementia patients. Collectively, these studies underscore the importance of incorporating physical exercise into care plans for dementia patients to enhance appetite and improve overall health.

Previous studies have shown similar benefits of structured activities on BPSD. For example, Barreto et al. (2015) demonstrated that exercise and behavioral management training could reduce depression in dementia patients (27). Similarly, Burley et al. (2022) found that non-pharmacological interventions, including cognitive stimulation and rehabilitation, effectively reduced BPSD symptoms (28). The present study’s findings align with these results, reinforcing the importance of multi-domain programs in managing BPSD.

However, it is noteworthy that while our study found significant improvements in appetite/eating distress (*p* = 0.026), the effect size was modest (mean change: −0.2), and other BPSD symptoms did not show significant changes. This discrepancy might be due to the specific components of our intervention or the duration of the program. In contrast, studies such as Brodaty et al. (2003) have reported broader improvements in BPSD symptoms with longer intervention durations and more intensive caregiver involvement (29). Our findings suggest that while short-term, focused interventions can yield specific benefits, a more extended and comprehensive approach may be necessary to see broader symptom relief.

The program’s impact on caregiver burden and depression was limited. While there was a trend toward reduced burden among family caregivers in the intervention group, as indicated by the change in ZBI scores (−3.1 vs. –1.2, *p* = 0.306), this difference did not reach statistical significance. Similarly, there was no significant difference in depression levels between the intervention and control groups, as measured by CES-D scores (−0.1 vs. –1.2, *p* = 0.7). These findings align with previous research that has shown mixed results in caregiver interventions. For instance, He et al. (2022) found that multi-component interventions addressing both practical caregiving skills and emotional support were more effective in reducing caregiver burden and depression (30). In contrast, other studies, such as a meta-analysis by van der Prick et al. (2015), have highlighted the challenges in achieving significant reductions in caregiver burden and depression, indicating the complex nature of these outcomes in dementia caregiving (31). While our intervention demonstrated some positive trends, it underscores the ongoing need for comprehensive and tailored approaches to effectively support caregivers of dementia patients.

The integration of digital interventions may offer a unique avenue for enhancing the impact of such programs. Knapp et al. (2022) discussed the potential for digital interventions to be effective, cost-effective, and equitable in managing dementia symptoms (32). Their findings suggest that digital platforms can provide accessible and scalable solutions for both patients and caregivers, although challenges remain in ensuring widespread and equitable access. Similarly, Di Lorito et al. (2022) conducted a systematic literature review and meta-analysis on digital health interventions, concluding that these interventions can be beneficial for people living with dementia and mild cognitive impairment (33). However, they also highlighted the need for further research to optimize these interventions and fully understand their long-term benefits and limitations. Moon and Park (2020) explored the effects of digital reminiscence therapy on people with dementia, finding positive outcomes in terms of engagement and symptom management (34). The present study used a video-based training program, which may be cost-effective and easily propagated between care sites for people with dementia.

The strengths of our study are the comprehensive nature of the intervention, its cluster-randomized design, and low drop-out rate (1.9%). This multi-domain approach likely contributed to the observed benefits. However, the study had limitations. First, selection bias may exist in the present study. The control and intervention CSCDs may not be comparable. Despite matching for severity, the intervention group had a slightly lower baseline severity of dementia compared to the control group (CDR = 0.5 72% vs. 55.2%, *p* = 0.013) and also lower caregiving burden rated by care staff in the CSCDs (ZBI pre-test 6.3 vs. 10.4, *p* = 0.008). This difference could have influenced the outcomes, suggesting that future studies should ensure even more rigorous matching or consider stratifying participants by severity to better isolate the intervention’s effects. Second, ascertainment bias may also exist. The study relied on self-reported measures; however, both raters were not blinded to the intervention. Third, the sample size, while sufficient for the initial findings, may limit the generalizability of the results.

In conclusion, the 16-week video-based multiple training modality intervention program demonstrated significant positive effects on specific neuropsychological symptoms in dementia patients, particularly in reducing appetite/eating distress. While the impact on overall BPSD was limited, the findings highlight the potential of structured and engaging activities to improve certain symptoms. However, the program’s impact on caregiver burden and depression was limited, indicating a need for more targeted support for caregivers. These findings underscore the importance of developing comprehensive and tailored interventions that address both the needs of dementia patients and their caregivers. Promoting the application of such video-based multiple training modalities to other CSCDs in the whole of Taiwan was recommended. The video-based intervention is designed to provide a replicable, structured, and empirically grounded program that can be uniformly delivered across multiple sites, meeting increasing demands for dementia care. Our study also provides a meaningful initial exploration of the potential role for video-based interventions in the context of increasing demand for dementia. Future studies should include larger patient groups, and more intensive interventions may be needed to reach a broader effect; nonetheless, the study demonstrates a potential role of video-based intervention meeting the increasing demand for dementia care.

## Data Availability

The raw data supporting the conclusions of this article will be made available by the authors, without undue reservation.
